# Mucosal vaccination with pili from Group A Streptococcus expressed on *Lactococcus lactis* generates protective immune responses

**DOI:** 10.1038/s41598-017-07602-0

**Published:** 2017-08-03

**Authors:** Jacelyn M. S. Loh, Natalie Lorenz, Catherine J.-Y. Tsai, Adrina Hema J. Khemlani, Thomas Proft

**Affiliations:** 1 Department of Molecular Medicine & Pathology, School of Medical Sciences, The University of Auckland, Auckland, 1023 New Zealand; 2 Maurice Wilkins Centre for Molecular Biodiscovery, Auckland, 1023 New Zealand

**Keywords:** Pathogens, Bacterial infection

## Abstract

The human pathogen Group A Streptococcus (GAS) produces pili that are involved in adhesion and colonisation of the host. These surface-exposed pili are immunogenic and therefore represent an attractive target for vaccine development. The pilus is encoded in the genomic region known as the fibronectin-collagen-T-antigen (FCT)-region, of which at least nine different types have been identified. In this study we investigate expressing two of the most common FCT-types (FCT-3 and FCT-4) in the food-grade bacteria *Lactococcus lactis* for use as a mucosal vaccine. We show that mucosally delivered *L*. *lactis* expressing GAS pili generates specific antibody responses in rabbits. Rabbit anti-pilus antibodies were shown to have both a neutralising effect on bacterial adhesion, and immunised rabbit antiserum was able to facilitate immune-mediated killing of bacteria via opsonophagocytosis. Furthermore, intranasal immunisation of mice improved clearance rates of GAS after nasopharyngeal challenge. These results demonstrate the potential for a novel, pilus-based vaccine to protect against GAS infections.

## Introduction

Group A Streptococcus or *Streptococcus pyogenes* is a major human pathogen that causes a range of diseases, from minor skin and throat infections such as impetigo and pharyngitis, to severe invasive infections such as streptococcal toxic shock syndrome and necrotising fasciitis^1–3^. Recurring skin and throat infections are common in developing countries, as well as in areas of low socioeconomic status within developed nations. There is also a clear link between GAS burden and the development of acute rheumatic fever (ARF) and rheumatic heart disease (RHD)^1–3^. These diseases carry significant morbidity and mortality globally, with an estimated incidence of 16–20 million cases/year^1–3^.

Despite decades of on-going research, a safe and effective vaccine to prevent GAS infections has not yet been realised. Numerous candidate vaccines however are starting to reach clinical trials, with the most advanced candidate passing phase II trials being the 26-valent M-protein-based vaccine^4^. This vaccine contains a fusion of recombinant N-terminal peptides from 26 different M-proteins^4^. However, recent studies have suggested that this vaccine might provide poor coverage of strains circulating in many developing countries or in low socio-economic regions of industrialised countries such as New Zealand and Australia^5–7^. M-protein-based vaccines have also in the past raised concerns due to potential cross-reactivity of antibodies to human proteins implicated in the development of rheumatic fever^8^.

The pilus of GAS represents an alternative non-M-protein-based vaccine target. Pili of GAS were first described in 2005 as long, flexible hair-like filaments that protrude from the bacterial surface^9^. They have since been shown to be involved in adhesion and colonisation of the host^10–12^. The major component of the pilus structure is the backbone protein (BP) also known as the T-antigen, of which 10–100 subunits are covalently linked to form fibres up to 10 µm long. Attached to either end are 1 to 2 accessory proteins (AP1 and AP2). AP1 often has adhesive properties, and AP2 often serves as an adapter protein for sortase-mediated cell wall anchorage^13^. The pilus and its assembly enzymes are encoded in the genomic region known as the FCT-region^14,15^. Nine different FCT-types have been described based on their gene composition and DNA sequence^16^. Systemic immunisation of mice with recombinant pilus proteins from FCT-2 has previously shown to confer protection against GAS challenge^9^. It has been suggested that generating a mucosal immune response may provide an added advantage in protecting against GAS infection^17,18^, since its major route of entry is via a mucosal site. The food-grade bacterium *L*. *lactis* provides an attractive vehicle for mucosal vaccine delivery as it is inexpensive to produce and does not require the use of toxic adjuvants. Previous studies have shown that *L*. *lactis* is able to express the pilus island 1 from Group B Streptococcus (GBS), and can protect mice from challenge with GBS isolates carrying this pilus^19^.

In this study we show the expression of the GAS pilus on the surface of *L*. *lactis* as a novel mucosal vaccine strategy against GAS infections. FCT-3 and FCT-4 are by far the most common FCT-types of GAS, covering approximately 70% of clinical isolates^15,20^. We have therefore chosen to clone and express the pilus operon from these two FCT-types to demonstrate the ability of recombinant *L*. *lactis* strains to elicit protective immune responses in this proof of concept study.

## Results

### The pilus from GAS can be expressed on the surface of *L*. *lactis*

**Figure 1:**
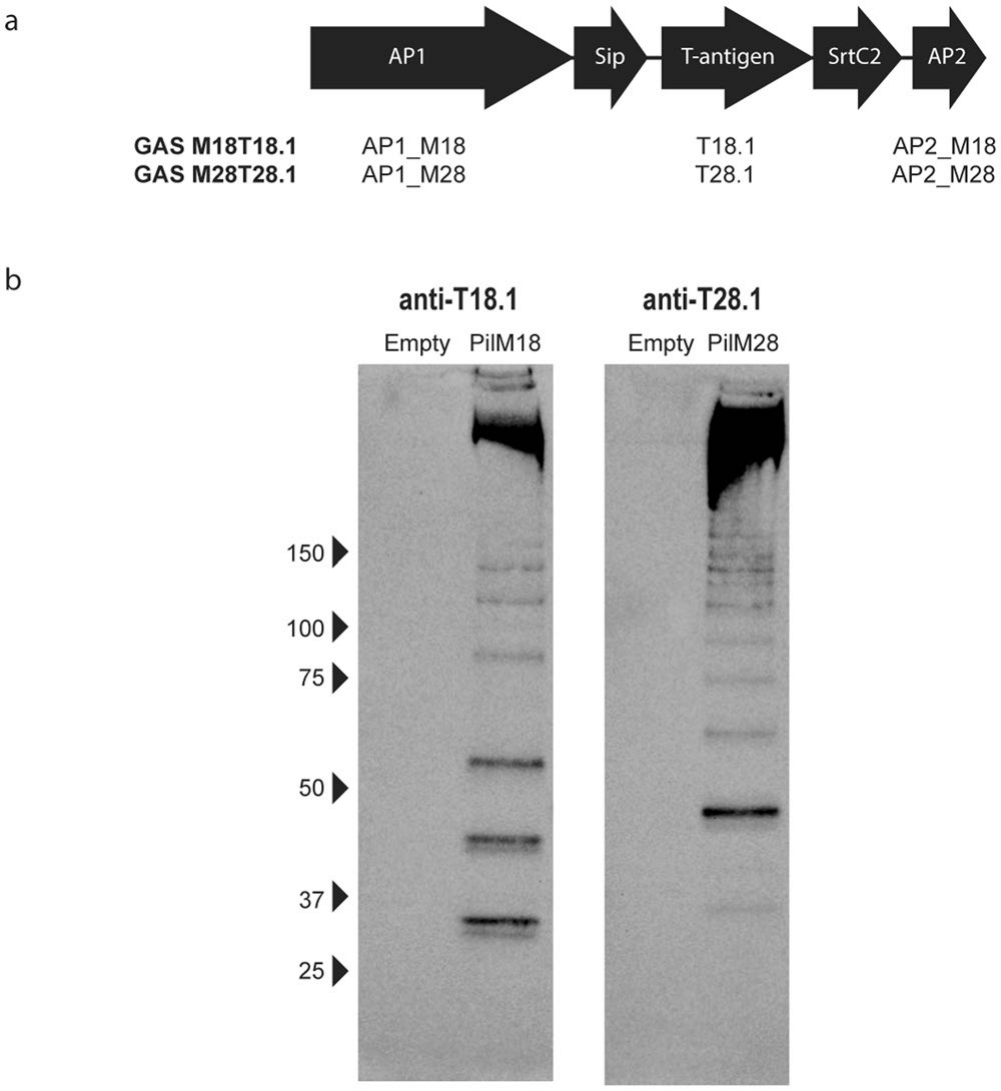
Cloning and expression of the GAS pilus on *L*. *lactis*. The entire GAS operon from GAS M18T18.1 and GAS M28T28.1 was cloned into the expression vector pLZ12-Km2 P23R and transformed into *L*. *lactis*. Genes that encode AP1, T-antigen, and AP2 are indicated in the schematic diagram of the operon (**a**). Cell wall extracts were prepared from the *L*. *lactis* clones, separated on a 4–15% gradient gel and blotted onto nitrocellulose membrane. Pilus expression was detected using rabbit polyclonal antibodies against T18.1 or T28.1 (**b**).

The pilus operon (Fig. 1a) was cloned from GAS M18T18.1 and GAS M28T28.1, and expressed in *L*. *lactis* under control of the constitutive lactococcal P23 promoter. These clones were named *L*. *lactis* PilM18 and *L*. *lactis* PilM28, respectively. Pilus expression on the surface of *L*. *lactis* was confirmed by Western blot analysis of cell-wall extracts from the respective *L*. *lactis* clones. Western blots show a high molecular weight laddering pattern from the PilM18 or PilM28 cell wall extracts using antibodies against the respective T-antigen, with most pili above 150 kDa (Fig. 1b). This laddering pattern is typical of pilus expression as each pilus structure can contain a variable number of covalently linked T-antigen subunits, thereby forming different length pili. No bands were visible from cell wall extracts of *L*. *lactis* carrying the empty vector as expected (Fig. 1b).

### Immunisation with *L*. *lactis* PilM18 or PilM28 generates specific antibody responses against the GAS pilus in rabbits

**Figure 2:**
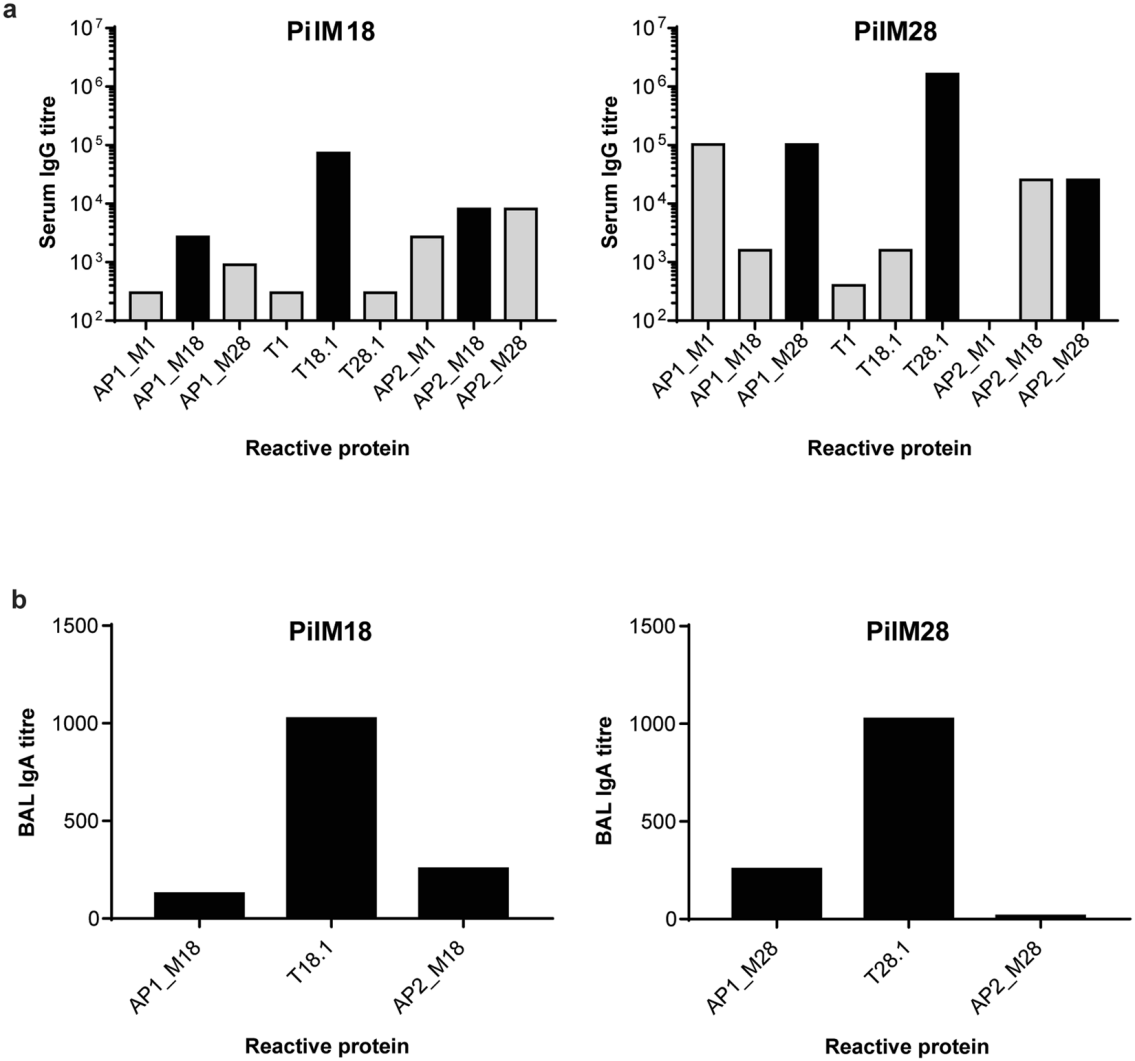
Rabbit antibody responses to oral immunisation with *L*. *lactis* expressing GAS pili. Serum (**a**) or BAL (**b**) antibody titres from rabbits immunised with *L*. *lactis* PilM18 or *L*. *lactis* PilM28 were determined by ELISA against purified recombinant proteins. Black bars indicate antibody titres against proteins present in the vaccine. Grey bars indicate antibody titres against cross-reactive antigens. Two technical replicates were performed.

Serum and BAL fluid from NZW rabbits immunised with *L*. *lactis* PilM18 or *L*. *lactis* PilM28 were analysed for their antibody response by ELISA (Fig. 2). Serum IgG was the highest against the T-antigen, and a stronger response was observed with PilM28 (titre = 10^6^) than PilM18 (titre = 10^5^). IgG titres against the 2 accessory proteins (AP1 and AP2) were approximately 10-fold lower than that of the T-antigen (Fig. 2a). IgA titres against all 3 pilus proteins were detectable in the BAL fluid and were the highest against the T-antigen (Fig. 2b), reflective of what was seen in the serum (Fig. 2a).

Cross-reactivity of the immunised rabbit serum was also analysed against individual pilus proteins of the alternate pilus, and against a more distantly-related pilus (of the FCT-2 type) from GAS M1T1 (Fig. 2a). Cross-reactivity was observed with PilM18 antiserum against pilus proteins from the M28 strain, predominantly against the AP2_M28 anchor protein. A lower cross-reactivity was also observed against AP2_M1. Similarly, cross-reactivity was observed with PilM28 antiserum against pilus proteins from the M18 strain, again predominantly against AP2_M18. Interestingly, PilM28 antiserum did not cross-react with AP2_M1, but did against the AP1_M1 tip adhesin.

**Figure 3:**
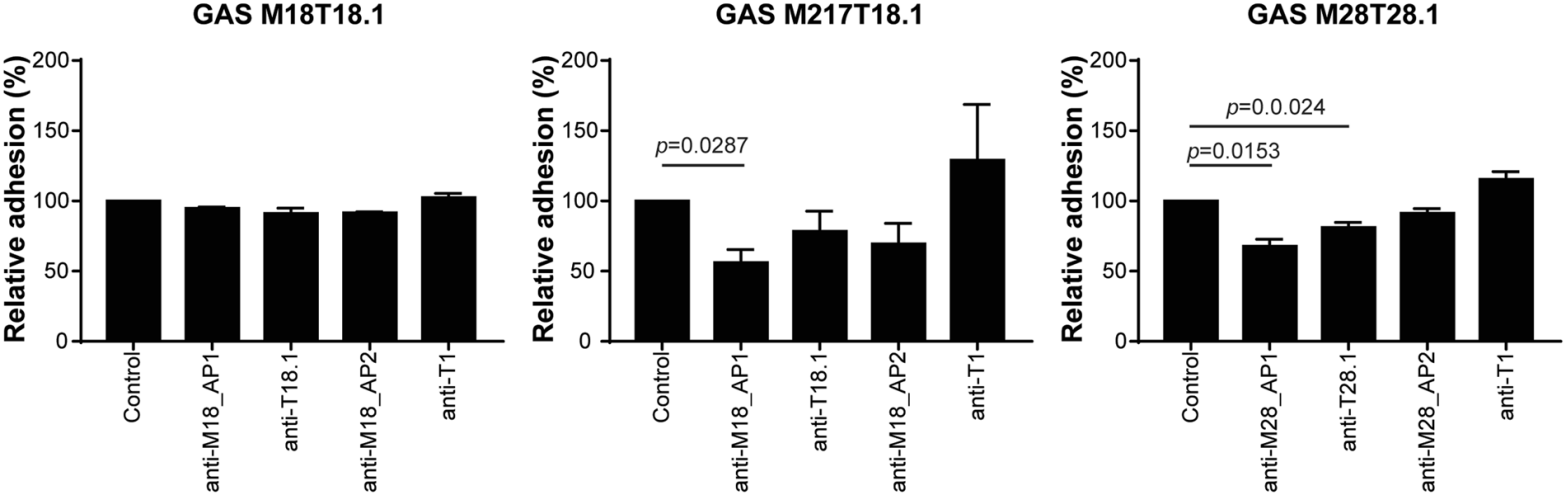
Antibodies against pilus proteins can neutralise adherence of GAS to HaCat cells. HaCat monolayers were incubated with indicated GAS strains for 1 h in absence (control), or presence of purified antibodies. Wells were washed to remove unbound bacteria and amount of bacteria assessed by bioluminescence (which is proportional to CFU). Adhesion was calculated as the proportion of bacteria remaining after washing compared to the unwashed wells. Data is expressed as the percentage of adhesion relative to the no antibody control (which was set to 100%). Bars indicate the mean ± SD of 3 biological replicates. Statistical analysis performed using one-way ANOVA.

Next we tested if the antibodies generated against the pili were functional. Pili are known to be involved in adhesion and hence colonisation of host tissue, we therefore first investigated if anti-pilus antibodies could neutralise adhesion. To do this, we used an immortalised human keratinocyte cell line (HaCat) to represent a common infection site of GAS (skin). This cell line was chosen over a pharyngeal cell line (Detoit562) because we have previously shown that the majority of GAS strains preferentially adhere to HaCat cells^21^. Adhesion of GAS M18T18.1 or GAS M28T28.1 to HaCat cell monolayers was observed. However, addition of antibodies raised against individual pilus proteins of the M18T18.1 strain failed to prevent this adhesion (Fig. 3). As this result was unexpected, we tested if adhesion could be inhibited on a closely related GAS strain (GAS M217T18.1) that carries the same pilus operon. Indeed, antibodies against the tip adhesin AP1_M18, significantly reduced adherence of this strain to HaCat cells by approximately 50% (p = 0.0287) (Fig. 3). Furthermore, both anti-AP1_M28 and anti-T28.1 antibodies were able to significantly reduce adherence of GAS M28T28.1 to HaCat monolayers by 30% (p = 0.0153) and 20% (p = 0.024), respectively (Fig. 3). As expected, non-specific antibodies against a distantly-related pilus type (anti-T1) were unable to reduce adherence.

**Figure 4:**
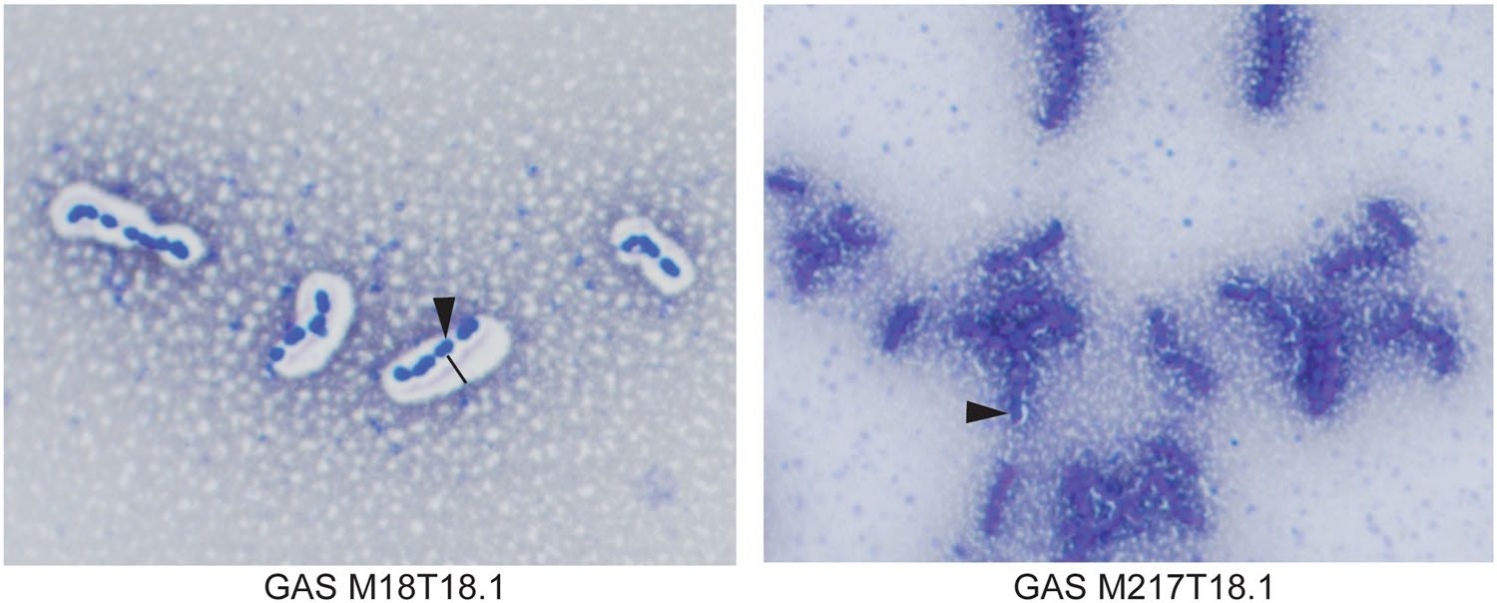
Capsule Staining of GAS. Bacterial smears were stained with Indian ink then counter-stained with crystal violet. Capsule is observed as a white halo surrounding GAS M18T18.1, but not GAS M217T18.1. Arrow points to an example of a bacterium, line indicates thickness of capsule.

A possible reason for the lack of neutralising activity of the pilus-specific antibodies against the M18T18.1 strain might be encapsulation of the bacteria. Hyper-encapsulation of GAS masks surface antigens, and is one of the mechanisms the bacterium can use to evade the immune system. Some strains are known to have a greater ability to perform this function. To test if hyper-encapsulation could be the reason for the inability of antibodies to block adhesion of the GAS M18T18.1 strain, we compared capsule size to its close relative GAS M217T18.1 using India ink staining. A clear contrast was observed between these two strains with GAS M18T18.1 showing a far bigger capsule than GAS M217T18.1 (Fig. 4).

**Figure 5:**
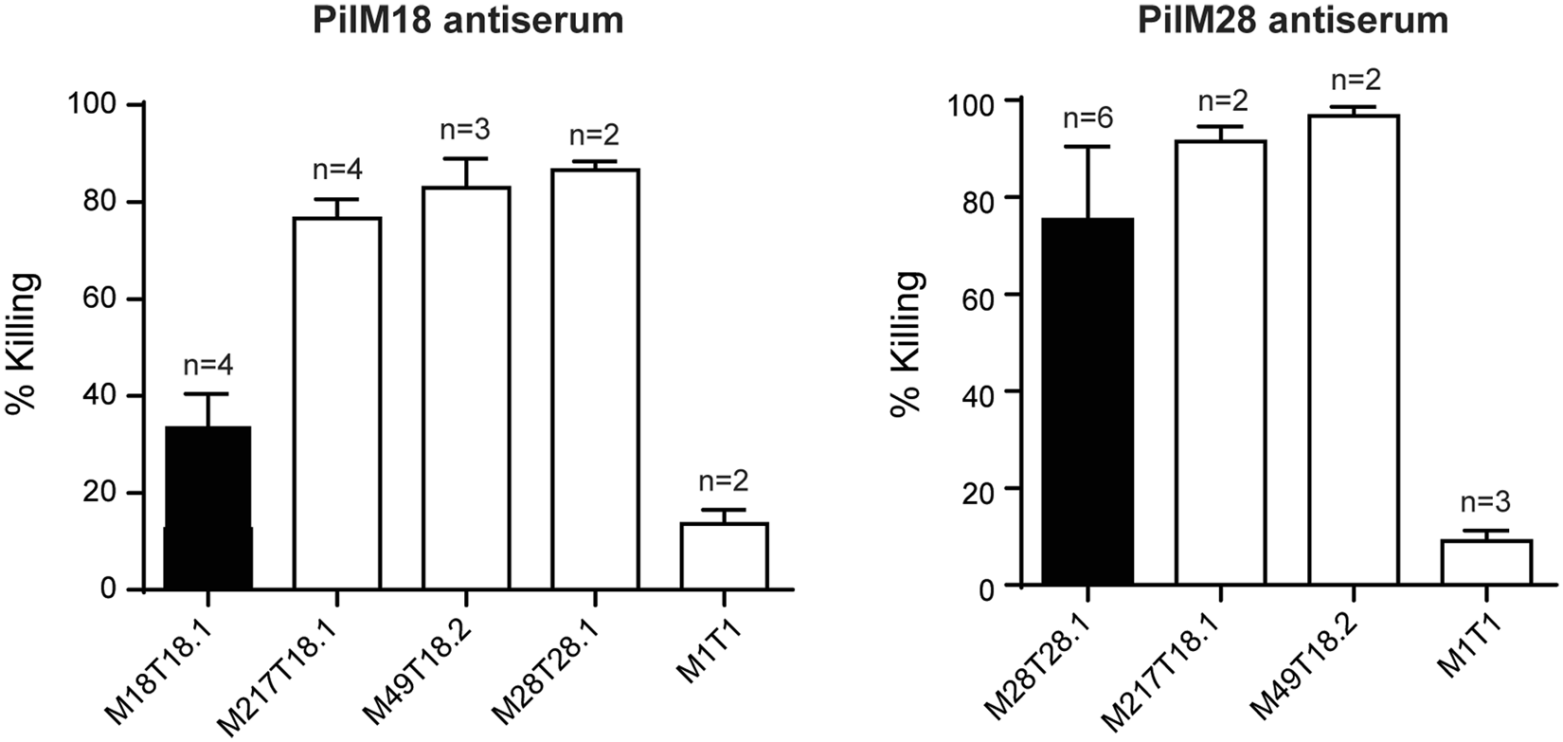
Immunised rabbit antisera have bactericidal activity against GAS. Sera from immunised rabbits were tested in an opsonophagocytosis assay against GAS strains as indicated. Bars indicate the mean ± SD of at least 2 biological replicates. Black bars indicate killing against the strain from which the vaccine was derived.

We next sought to determine if PilM18 or PilM28 antiserum was able to mediate bacterial killing through opsonophagocytosis. Specific killing was not observed with PilM18 antiserum against its respective strain GAS M18T18.1 (Fig. 5), most likely due to the hyper-encapsulation of this strain (Fig. 4). This was in line with the results from the neutralisation experiments. However, when testing this antiserum against the closely related, but less encapsulated strain GAS M217T18.1, over 70% killing was observed (Fig. 5). Cross-protection was also observed when PilM18 antiserum was tested against strains that expressed related T-antigens, GAS M49 (M49T18.2) and GAS M28 (M28T28.1). Killing of a distinct strain GAS M1 (M1T1) was not observed (Fig. 5). Similarly, PilM28 antiserum was able to mediate killing of GAS M28T28.1, GAS M217T18.1, and GAS M49T18.2, but not GAS M1T1 (Fig. 5).

### Immunisation with *L*. *lactis* PilM89 protects against nasopharyngeal colonisation in mice

**Figure 6:**
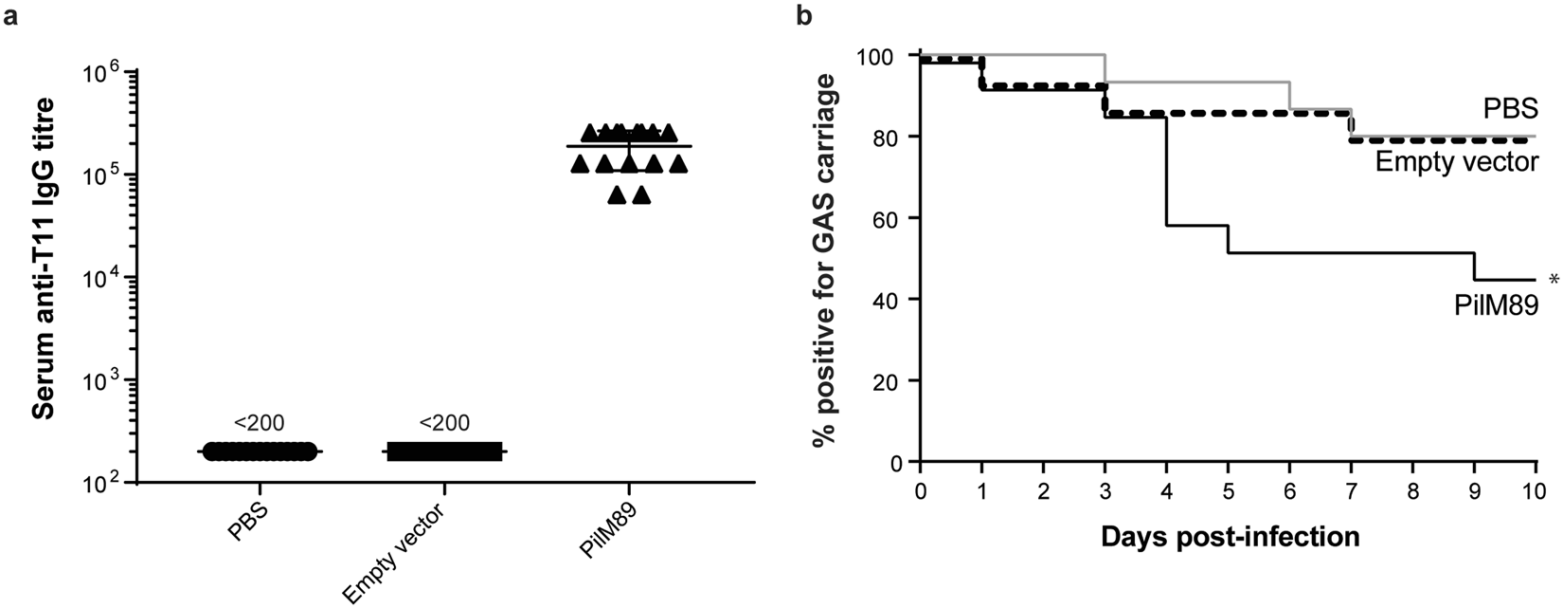
Intranasal immunisation of mice protects against nasopharyngeal challenge. FVB/n mice (n = 15) were immunised intranasally with PBS, *L*. *lactis* empty vector, or *L*. *lactis* PilM89. Serum was collected 10 days after the final immunisation and antibody responses measured against recombinant T11-antigen by ELISA (**a**). Mice were subsequently challenged intranasally with 10^8^ CFU GAS M89T11 and monitored for nasal shedding of bacteria daily. The percentage of mice positive for at least 1 CFU of GAS is displayed (**b**). Data combines two independent experiments. *p < 0.05, Logrank test for trend.

Initial tests to determine nasopharyngeal colonisation with various GAS strains showed that the majority of strains were poor murine colonisers, and were rapidly cleared from mice (Supplementary Figure 1). One of the best colonising strains we found was GAS M89T11 which carries the FCT-4 locus. For this reason, the pilus operon from GAS M89 was subsequently cloned and expressed in *L*. *lactis* (*L*. *lactis* PilM89). Mice immunised intranasally with *L*. *lactis* PilM89 produced high antibody titres against the T11-antigen (Fig. 6a) and showed a higher clearance rate when infected with GAS M89T11 compared to the controls (Fig. 6b). Fifty percent of mice immunised with *L*. *lactis* PilM89 had cleared the infection by day 5, compared to a clearance rate of approximately 20% in mice immunised with PBS or *L*. *lactis* carrying an empty vector (Fig. 6b).

## Discussion

Thus far, 9 different FCT regions have been identified in GAS. In this study we have selected the two most common pilus types (encoded in the FCT-3 and FCT-4 regions) to show proof-of-concept for their potential use as a vaccine against GAS. These pili were successfully expressed on the surface of the non-pathogenic bacterium *L*. *lactis*, and the resulting recombinant *L*. *lactis* can be used as a mucosal vaccine to generate functional antibodies against GAS without the addition of toxic adjuvants. Immunisation of rabbits via oral gavage produced both IgA and IgG against the 3 major pilus proteins. Response was the highest against the T-antigen in both cases, most likely due to this subunit’s abundance in the multimeric structure of the pilus shaft, while AP1 and AP2 are present as single subunits at the tip and base of the pilus, respectively. The FCT-3 and FCT-4 encoded pili are not only the most common, but also the most closely related pili based on amino acid sequence identities of the individual pilus proteins (Supplementary Figure 2). Cross-reactivity against all 3 pilus proteins was observed, with the highest occurring against the AP2 protein. This is perhaps not surprising given that the AP2 from both strains have a 96% amino acid sequence identity (Supplementary Figure 2). Antiserum was also tested against a more distantly-related pilus from the GAS M1T1 strain. Although both AP2_M18 and AP2_M28 share only 33% and 28% amino acid sequence identity with AP2_M1, respectively, cross-reactivity was observed against AP2_M1 with the PilM18 antiserum but not PilM28 antiserum. This suggests that an antibody against a similar structural epitope is found in the PilM18 antiserum that is not present in the PilM28 antiserum. It is notable that T-antigens and AP2 proteins share common IgG-like folds^22–24^ despite low sequence identity. High cross-reactivity was observed against AP1_M1 when testing the PilM28 antiserum, suggesting a shared antigenic epitope. Sequence identity between the AP1_M1 and AP1_M28 is 60%, while sequence identity between AP1_M1 and AP1_M18, or between AP1_M28 and AP1_M18 is 54%.

It was hypothesised that antibodies generated against AP1 would likely have a neutralising function, as AP1 is the known adhesin of the pilus structure. Using adhesion assays with HaCat cell monolayers, it was shown that this was indeed the case. Anti-AP1_M28 was able to significantly reduce the adherence of GAS M28T28.1 to HaCat monolayers. Although anti-AP1_M18 antibodies were unable to block the adherence of GAS M18T18.1, it was shown that GAS M18T18.1 is hyper-encapsulated, therefore probably masking the surface antigens in this strain. This is supported by the fact that anti-AP1_M18 antibodies were able to significantly reduce the adherence of a closely related but minimally encapsulated strain, GAS M217T18.1.

It was also observed that anti-T28.1 antibodies were able to reduce adherence of GAS M28T28.1, albeit to a lesser extent than anti-AP1_M28 antibodies, suggesting perhaps an unexpected role in adhesion for this T-antigen. Although not typically known for its role in adhesion, the T-antigen has been shown to be involved in adhesion in some strains of GAS. For example, the T2-antigen, not the AP1 protein, of GAS M2 (MGAS10270) has been shown to be the main adhesin of the FCT-6 pilus^25^.

While antibodies against the pilus can contribute to the reduction of adherence, complete reduction is not achieved. This is expected as GAS is known to express other adhesive molecules. GAS expresses a number of fibronectin binding proteins, collagen-like proteins, and the M-protein, all known to be involved in adhesion (reviewed in ref.^26^).

In addition to neutralising antibodies, it was hypothesised that the vaccine could mediate the killing of bacteria by generating opsonising antibodies. Using an *in vitro* opsonophagocytosis assay, we saw antibody-mediated killing against GAS strains from which the pilus was derived, as well as cross-protection against strains that expressed closely-related pili. The exception was that the GAS M18T18.1 strain was not killed with the PilM18 antiserum, though this was likely due to the presence of the large capsule preventing antibodies from opsonising.

Our results cannot determine whether it was the antibodies directed against AP1, T-antigen, AP2, or a combination of these antibodies that was most effective for immune-mediated killing. Although by ELISA, cross-reactivity was observed to AP2_M18 with the PilM28 antiserum, antibodies against AP2 would not be expected to play a major role due to its location at the very base of the pilus structure, hence its reduced accessibility to immune-mediated responses. Cross-reactive antibodies to AP1_M1 were also observed with the PilM28 antiserum by ELISA; however, the GAS M1T1 strain could not be killed in our assay. We therefore hypothesise that the T-antigen is most likely the major target for opsonophagocytotic killing by our antisera. Indeed, the highest antibody titre was generated against the T-antigen in our animals.

Sequence analysis of 57 GAS strains revealed 15 *bp*/*tee*, 16 *ap1* and 5 *ap2* sequence variants^16^. This was later expanded to 18 *bp/tee* variants when analysing 100 GAS strains^20^. The extent of cross-protection to other GAS strains would therefore require assessment in the future to optimise the formulation of a vaccine that would provide broad coverage of the majority of GAS strains.

To determine the protective capacity of our vaccine *in vivo* we chose a murine model of nasopharyngeal infection. The nasopharynx is a primary site of GAS infection and colonisation in humans and would most likely benefit from a mucosal vaccine. Initial set-up of this model showed that the majority of GAS strains tested had poor infection/colonisation rates in mice (Supplementary Figure 1) similar to that seen in previous studies^27^. Nevertheless, two strains (GAS M89T11 and GAS M49T18.2) were observed to be good colonisers, with GAS M89T11 showing the highest infection rates. The pilus operon from this strain was therefore subsequently cloned into *L*. *lactis* and used to intranasally immunise mice. Serum antibody levels to the T-antigen were high, and clearance rate after GAS M89T11 infection was higher than in mice immunised with *L*. *lactis* empty vector or PBS. When analysing individual T11-responding mice, there was no apparent correlation between higher serum antibody levels and a faster clearance rate of GAS infection. However, a limitation of this study was that local immune responses were not measured, which may have provided better correlation.

The use of *L. lactis* as a vector for delivering mucosal vaccines has been studied for decades with very promising results (reviewed by Wells *et al*.^28,29^). As a non-pathogenic, non-colonising species that has been consumed by humans for centuries, *L*. *lactis* has an extremely good safety profile. However, important questions surrounding the immunological responses to the vector itself remain to be addressed. Although reported to have low innate antigenicity, elevated antibody titres to native *L*. *lactis* antigens have been described post-administration^30^. These may be beneficial by providing adjuvanticity to the vaccine antigen, or potentially unfavourable if promoting tolerance. However, the nature of the vaccine antigen, the dose, and the route of administration will all play a role in the type of immune response generated.

In conclusion, our results demonstrate the potential for a novel, pilus-based vaccine to protect against GAS infections.

## Methods

### Animal Ethics

All animal experiments were performed in the Vernon Jansen Unit (University of Auckland, New Zealand) in accordance with relevant guidelines and regulations approved by the University of Auckland Animal Ethics Committee.

### Bacteria and cell culture conditions

GAS strains used in this study are listed in Supplementary Table 1. Strains were cultured in brain heart infusion medium (BHI) at 37 °C under static conditions. Strains were bioluminescently labelled as previously described^31^. *L*. *lactis* MG1363 was cultured in M17 medium supplemented with 0.5% glucose and 200 µg/ml kanamycin where required. BL21(DE3)pLysS *E*. *coli* (Novagen) was cultured in LB at 37 °C, 200 rpm with 30 µg/ml chloramphenicol and 50 µg/ml ampicillin where required.

### Cloning and protein expression

The complete pilus operon, including 5 open reading frames (Fig. 1a), was amplified from genomic DNA by PCR, cloned into the pLZ12-Km2 P23R vector^31^ downstream of the constitutive P23 lactococcal promotor, and electroporated into *L*. *lactis*. To generate recombinant proteins of individual pilins, the regions between the predicted N-terminal signal peptide sequence and the C-terminal cell wall anchor motif were amplified by PCR, cloned into the expression vector pET32a3c, and transformed into BL21(DE3)pLysS *E*. *coli*. Proteins were expressed as previously described^32^ and purified by immobilised-metal affinity chromatography using NTA-Ni^2+^ resin according to manufacturer’s instructions (Bio-Rad). Primers used in this study are listed in Supplementary Table 2.

### Cell wall extracts and Western blots

Cell wall extracts and Western blots were performed as previously described^25^. Western blots were probed with polyclonal rabbit anti-T18.1 or anti-T28.1 antibodies (produced in-house), then goat anti-rabbit IgG-HRP (Santa Cruz Biotechnology). Detection was performed using Amersham ECL Prime Western blotting detection reagent (GE Healthcare) and a ChemiDoc™ Imaging System (Bio-Rad).

### Immunisation

For generation of polyclonal antiserum against individual pilus proteins, recombinant protein (50 µg for M18_AP1, 100 µg for all others) was injected subcutaneously with IFA (1:1) into New Zealand white (NZW) rabbits on days 0, 14, and 28. Serum was collected on day 42 and antibodies were affinity-purified on CNBr-coupled sepharose (GE Healthcare) according to manufacturer’s instructions. For *L*. *lactis* immunisation, aliquots of bacteria were frozen in PBS/10% glycerol and enumerated by plating. On immunisation days, an aliquot was thawed at RT, washed, and resuspended in 0.5 ml PBS. Bacteria were heat-killed (as our ethics did not allow for use of live GMO bacteria) by incubating at 65 °C for 30 min, resuspended in 5 ml PBS and given by oral gavage to NZW rabbits. 5 × 10^9^ CFU *L*. *lactis* were administered on 3 consecutive days, 2 weeks apart, for a total of 9 doses (days 0, 1, 2, 14, 15, 16, 28, 29, 30). Pre-immune serum was collected on day 0 and immune serum was collected on day 42. Bronchoalveolar lavage (BAL) was performed with 5 ml PBS subsequent to exsanguination.

### ELISA

MaxiSorp plates (Nunc) were coated with 1 µg/ml recombinant protein in PBS overnight at 4 °C, prior to incubation with titrated serum. Detection was performed using goat anti-rabbit IgG-HRP or goat anti-mouse IgG-HRP (Santa Cruz Biotechnology) and 3,3,5,5-tetramethylbenzidine (ThermoFisher). Absorbance was determined on an EnSpire multilabel plate reader (Perkin Elmer). Endpoint titres were determined as the minimum serum dilution above the control (absorbance of 1:200 dilution of pre-immune serum +3 times the standard deviation).

### Adhesion assays

HaCat cells were cultured in Dulbecco’s Modified Eagle Medium/10% foetal bovine serum. Adhesion of GAS strains to HaCat cell monolayers was measured as previously described^25^ using overnight cultures of bioluminescently-labelled GAS. Antibodies to GAS pilus proteins were added at 10 µg/ml where indicated. Bacteria were detected by adding Luciferin (Gold Biotechnology) to a final concentration of 50 µg/ml, and bioluminescence (BLU) was measured on a VICTOR × 2030 multilabel plate reader (Perkin Elmer). Percentage adherence was calculated as BLU_washed_/BLU_unwashed_ × 100. Data is expressed as the percentage of adhesion relative to the no antibody control (which was set to 100%). Statistical analysis was performed using GraphPad Prism software.

### Killing assays

A 3 h *in vitro* opsonophagocytosis assay was performed in white 96-well plates as previously described^33^. Bioluminescence was measured on an EnSpire multilabel plate reader. Percentage killing was calculated as (BLU_pre-immune serum_ − BLU_immune serum_)/BLU_pre-immune serum_ × 100.

### Capsule staining

Early exponential phase bacteria were mixed with Indian ink and smeared onto a microscope slide and allowed to air dry. The slide was then stained with crystal violet for 1 min then rinsed with water. Slides were examined microscopically at 1000x magnification on a Nikon Eclipse E600 microscope for the presence of capsule, as indicated by clear zones surrounding the cells.

### Murine nasopharyngeal challenge

Five to six week old FVB/n mice (n = 5–10) were immunised under isoflurane anaesthesia intranasally with 10^8^ CFU live recombinant *L*. *lactis* or PBS control in a 5 µl volume on 3 consecutive days. Additional boosters were given, 2 weeks apart, for a total of 12 doses. Ten days after the final immunisation mice were given a nasopharyngeal challenge similar to previously described^27^. Mice were infected with 10^8^ CFU/5 µl/mouse of exponential phase GAS M89T11 and monitored daily for nasal shedding. Monitoring was performed, similarly to that described previously^27^, by gently pressing the nares of each mouse onto the surface of a Columbia horse-blood agar plate ten times and culturing the plates overnight at 37 °C. Mice were scored as having cleared the infection if no β-haemolytic colonies were cultured for at least 2 consecutive days.

## Supplementary information

Supplementary Data

## Data Availability

The datasets generated during and/or analysed during the current study are available from the corresponding author on reasonable request.
