# Publisher Correction: Mucosal vaccination with pili from Group A Streptococcus expressed on *Lactococcus lactis* generates protective immune responses

**DOI:** 10.1038/s41598-019-43123-8

**Published:** 2019-05-01

**Authors:** Jacelyn M. S. Loh, Natalie Lorenz, Catherine J.-Y. Tsai, Adrina Hema J. Khemlani, Thomas Proft

**Affiliations:** 1 Department of Molecular Medicine & Pathology, School of Medical Sciences, The University of Auckland, Auckland, 1023 New Zealand; 2 Maurice Wilkins Centre for Molecular Biodiscovery, Auckland, 1023 New Zealand

Correction to: *Scientific Reports* 10.1038/s41598-017-07602-0, published online 03 August 2017

The supplementary dataset was omitted from the original version of this Article. This has been corrected in the HTML version of the Article; the PDF version was correct at the time of publication.

