# Supplementary material for: Mucosal vaccination with pili from Group A Streptococcus expressed on Lactococcus lactis generates protective immune responses
Source: Sci Rep. 2017 Aug 3;7:7174. doi: 10.1038/s41598-017-07602-0 (PMC5543120; doi:10.1038/s41598-017-07602-0)
Supplement: Supplementary file 1 — Supplementary Data [file 41598_2017_7602_MOESM1_ESM.pdf]

Supplementary Data

Mucosal vaccination with pili from Group A Streptococcus expressed on *Lactococcus lactis* generates protective immune responses

Jacelyn M. S. Loh[1,2], Natalie Lorenz[1,2], Catherine J-Y. Tsai[1,2], Adrina Hema J. Khemlani[1,2], and Thomas Proft[1,2*]

[1]Department of Molecular Medicine & Pathology, School of Medical Sciences, University of Auckland, Auckland 1023, New Zealand.

[2]Maurice Wilkins Centre for Molecular Biodiscovery, Auckland 1023, New Zealand.

*corresponding author email: t.proft@auckland.ac.nz

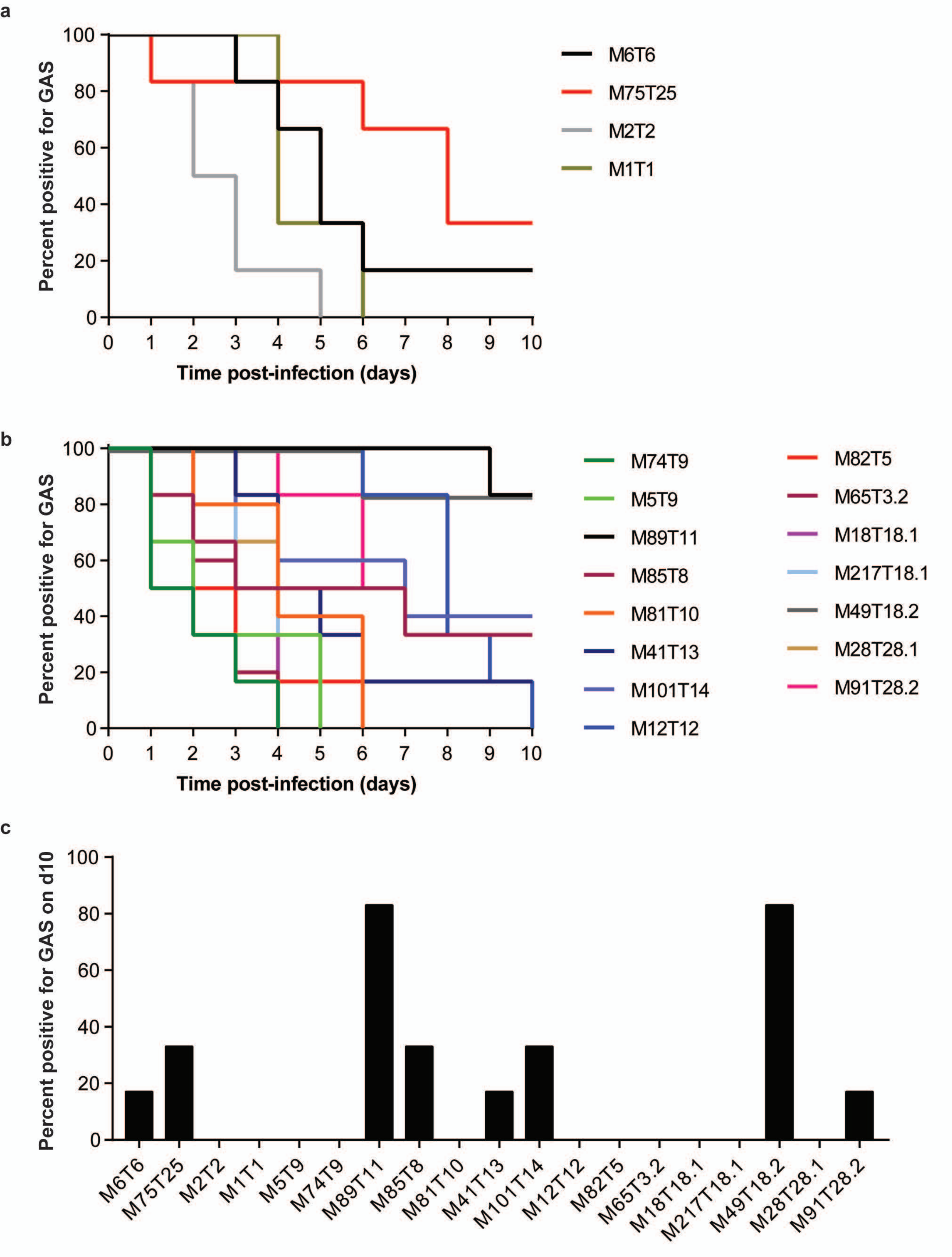

Supplementary Figure 1. Mouse infection studies.

FVB/n mice aged 10-12 weeks were challenged intranasally with 10^8 CFU of GAS (n=6). Strain information can be found in Table S1. Mice were monitored daily for nasal shedding of bacteria by culture on Columbia horse-blood agar plates. Graphs show the percentage of mice with a positive β-hemolytic culture of at least 1 CFU. A) Strains belonging to the FCT-1, 2, 5, and 6 types. B) Strains belonging to the FCT-3, 4, 7 or 8 types. C) Percentage of mice positive for GAS on day 10.

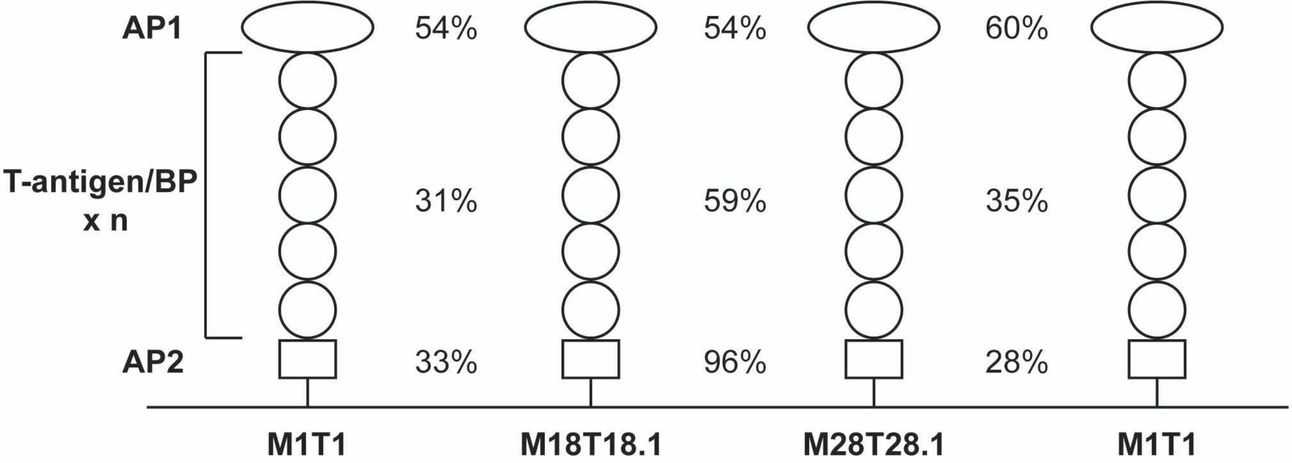

Supplementary Figure 2. Amino acid sequence identities between individual pilus proteins from different strains.

Amino acid sequences of mature proteins (from signal peptide cleavage site to sortase motif) were analysed for sequence identity using CLUSTALW.

Supplementary Table 1. Strains used in this study

Name	Strain	FCT-type	Origin
M6T6	MGAS10394	1	ATCC
M1T1	SF370	2	ATCC
M5T9	90/306 (LN85)	3	ESR
M41T13	10028 (LN79)	3	ESR
M12T12	94/354 (LN97)	3	ESR
M18T18.1	MGAS8232	3	ESR
M217T18.1	11574 (LN7)	3	ESR
M49T18.2	G (LN57)	3	ESR
M89T11	C (LN53)	4	ESR
M82T5	9893 (LN3)	4	ESR
M28T28.1	MGAS6180	4	ESR
M91T28.2	GAS09212 (LN115)	4	ESR
M85T8	GAS13256 (LN186)	3/4	ESR
M65T3.2	GAS1142 (LN127)	3/4	ESR
M74T9	GAS13345 (LN197)	3/4/7	ESR
M81T10	GAS10233 (LN122)	3/4/7	ESR
M101T14	GAS09426 (LN117)	3/4/7	ESR
M2T2	MGAS10270	6	ATCC
M75T25	GAS13232 (LN182)	9	ESR

ATCC, American Type Culture Collection. ESR, Institute of Environmental Science and Research New Zealand.

Supplementary Table 2. Primers used in this study

Product name	Primer	DNA sequence (5′–3′)
PilM18	PilM18.fw	gc**ggatcc**gaccatatacacggttgcagga
	PilM18_M28.rv	cgg**ctgcag**tcttatatggtacaaaaacctatcagc
PilM28	PilM28.fw	gc**ggatcc** tcttgacaataacgtggtaagctc
	PilM18_M28.rv	cgg**ctgcag**tcttatatggtacaaaaacctatcagc
AP1_M1	Spy0125.fw	**ggatcc**aagactgtttttggtttag
	Spy0125_LPXTG.rev	**ctcgag**ctaaccagtttctggcaaaggctctttattattttc
AP1_M18	spyM18_0126T53F	**ggatcc**actaaaaaaacgtcagtcattattag
	spyM18_0126I368R	**gaattc**ttaaataataggagcctgtttatcttc
AP1_M28	spyM28_0107.fw	ctag**ggatcc**gctcacgaattggttgagg
	spyM28_0107.rv	ctag**gaattc**ttaaaaaccagttggtgggacaag
T1	spy0128.fw	cg**ggatcc**gctacaacagttcacgg
	spy0128.rv	cg**gaattc**ttattcaaagactttttatttg
T18.1	spyM18_0128.fw	cg**ggatcc**gagacagcaggagtgattg
	spyM18_0128.rv	cg**gaattc**ttattcgtcagtagatttg
T28.1	spyM28_0109.fw	gc**ggatcc**gagacggcaggagtgtccgaaaatg
	spyM28_0109.rv	cg**gaattc**tta aacaccagttggaacttgagtg
T11	M89_FctAC.fw	gc**ggatcc**tctttagttgttaaaaaagagg
	M89_FctAC.rev	gc**gaattcaagatct**agttggaacttgagtgtcac
AP2_M1	Spy0130.fw	**ggatcc**gagaacctcactgcaagc
	Spy0130.rev	**gaattc**ttaaccattacgtttttctg
AP2_M18	spyM18_0130.fw	cg**ggatcc**aaagacagcactgttc
	spyM18_0130.rv	cg**gaattc**ttaggttttagggatgtcag
AP2_M28	spyM28_0111.fw	ctag**ggatcc**aaagacagcactgttcaaac
	spyM28_0111.rv	ctag**gaattc**ttattcaccagctaatggtaatg

Restriction sites are shown in bold.
